# Horner’s syndrome caused by the first rib fracture sustained during coronary artery bypass grafting: a case report and literature review

**DOI:** 10.1186/s44215-024-00166-2

**Published:** 2024-09-10

**Authors:** Hiroto Yasumura, Koji Tao, Ryo Imada, Yushi Yamashita, Naoki Tateishi, Tamahiro Kinjo

**Affiliations:** Department of Cardiovascular Surgery, Kagoshima Medical Center, 8-1, Shiroyamacho, Kagoshima, Kagoshima 892-0853 Japan

**Keywords:** Horner’s syndrome, Rib fracture, Open heart surgery, Coronary artery bypass grafting

## Abstract

**Background:**

Horner’s syndrome is a rare complication of cardiovascular surgery. A bone fragment and hematoma due to rib fracture after cardiac surgery may cause injury to the brachial nerve plexus and sympathetic nerve trunk, leading to neurologic disorders and Horner’s syndrome. However, few reports have revealed the etiology of Horner’s syndrome after cardiovascular surgery based on imaging. Herein we present a case in which a plain CT scan confirmed the etiology of Horner’s syndrome after coronary artery bypass grafting (CABG), reviewing 139 CABG cases retrospectively in our hospital and 6 case reports of Horner’s syndrome associated with cardiovascular surgery.

**Case presentation:**

A 69-year-old woman with a history of percutaneous coronary intervention and total abdominal hysterectomy with bilateral salpingo-oophorectomy had chest pain on exertion. Coronary angiography showed severe triple vessel disease. She underwent off-pump coronary artery bypass grafting (CABG). A median sternotomy was performed, and the split sternums were widened using a sternal retractor. The bilateral internal thoracic arteries were harvested. A triple CABG was performed. She had left shoulder pain after surgery. She complained of anhidrosis involving the left face and hyperhidrosis involving the right face from postoperative day (POD) 6. Left ptosis and blurry vision appeared after discharge from the hospital, for which she saw a neurologist in our hospital on POD 48. Miosis could not be clearly confirmed. She was diagnosed with Horner’s syndrome. A plain CT scan revealed displaced fractures of the bilateral first ribs and left second rib. The bone fragment of the left first rib head was displaced 3 mm anteriorly compared to the left first rib head before surgery, which suggested that the fragment affected the stellate ganglion in the sympathetic trunk. The patient had regular follow-up evaluations. The anhidrosis persisted, but the ptosis improved, and the miosis was not confirmed at the 6-month follow-up evaluation.

**Conclusions:**

We should recognize that Horner’s syndrome is one of the complications of cardiovascular surgery, especially CABG. Fracture of the first rib head with a displaced bone fracture was shown to be a contributor to ipsilateral Horner’s syndrome. When symptoms of Horner’s syndrome and other neurologic symptoms are noted after open heart surgery, a plain CT examination should be obtained.

## Background

Horner’s syndrome is a rare complication of cardiovascular surgery. The triad of Horner’s syndrome is ptosis, miosis, and anhidrosis, which leads to a lower aesthetic, visual, and hygienic quality of life [1]. The mean force of median sternotomy is a remarkably forceful procedure, requiring forces from 150 to 300 N (kg・m/s^2^) in corpses [2] and leading to rib fracture. A bone fragment and hematoma due to rib fracture may cause injury to the sympathetic nerve trunk and brachial nerve plexus [3, 4], leading to Horner’s syndrome and other neurologic disorders. However, few reports have revealed the etiology based on imaging. Herein we present a case in which a plain CT scan confirmed the etiology of Horner’s syndrome after coronary artery bypass grafting (CABG), reviewing 139 CABG cases retrospectively in our hospital and 6 case reports of Horner’s syndrome associated with cardiovascular surgery.

## Case presentation

**Fig. 1:**
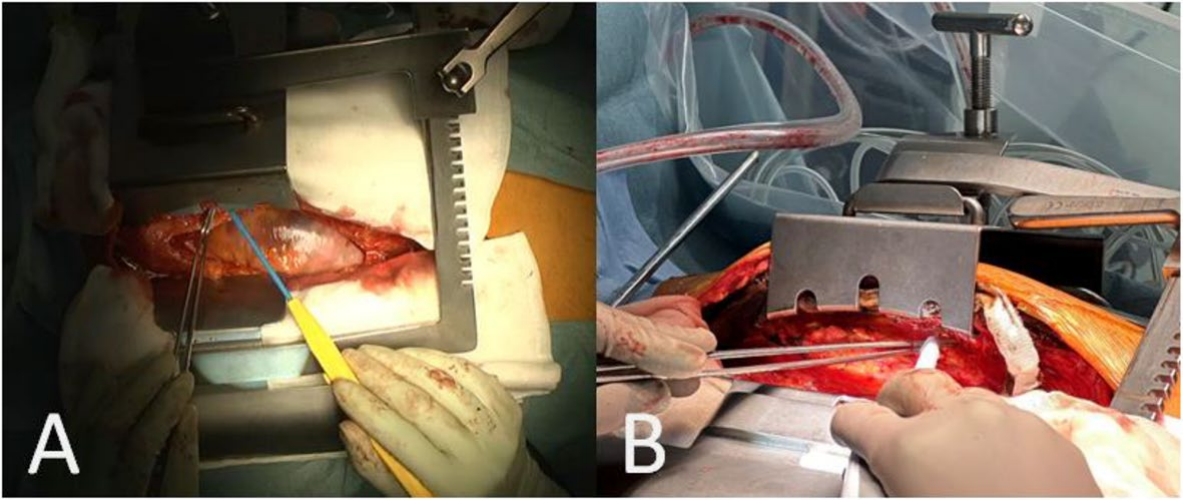
Finding of LITA harvesting (These are pictures of reference and irrelevant to the patient described herein). **A** A median sternotomy was performed and the split sternums were widened using a sternal retractor (IMR15-710-J; Getinge, Gothenburg, Sweden). The maximum chest opening width of this retractor is 130 mm. This is a picture of reference and irrelevant to the patient described herein. **B** Rib retraction can be adjusted by a screw. The maximum movement range of the screw is 70 mm

**Fig. 2:**
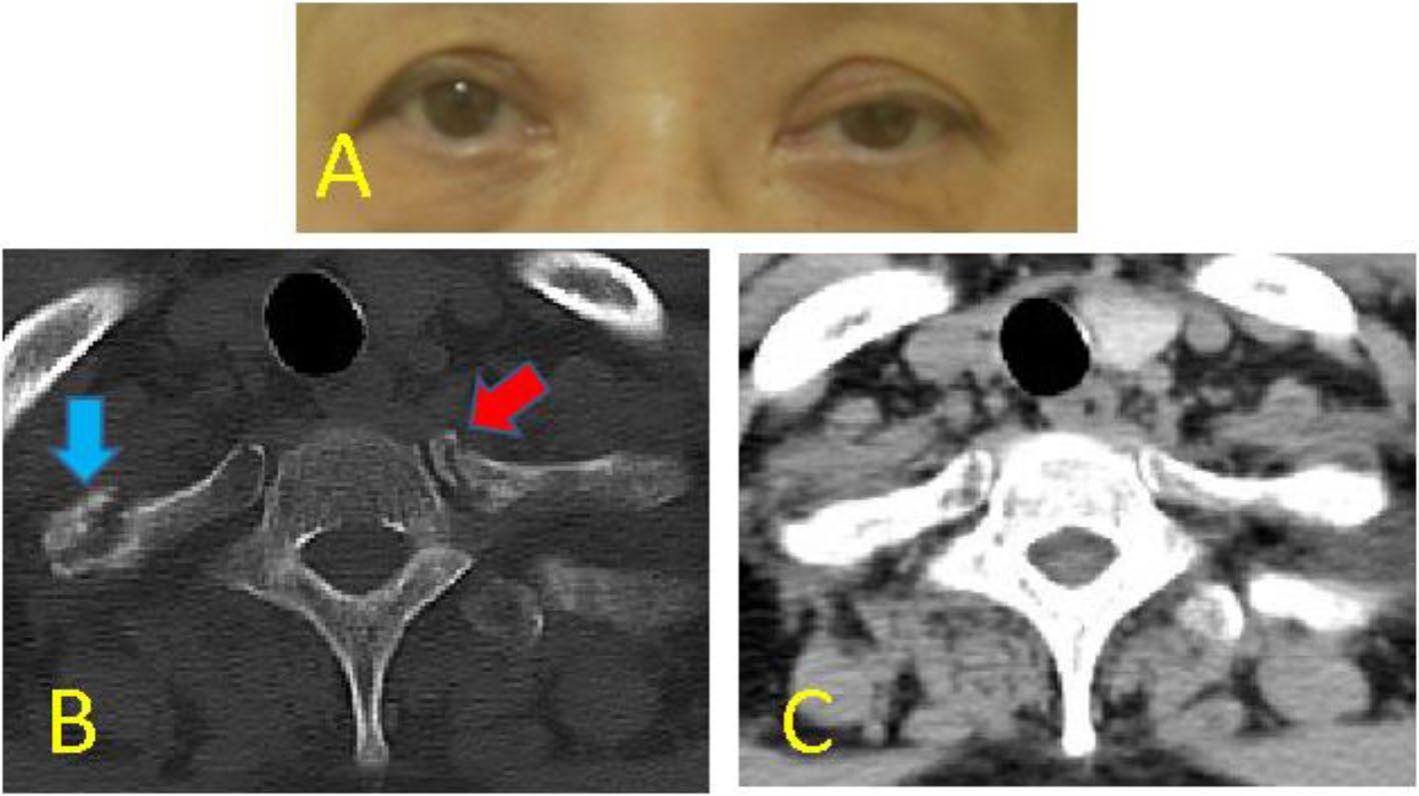
Findings before and after CABG. **A** Left ptosis appeared after discharge and she was diagnosed with Horner’s syndrome on postoperative day (POD) 48. **B** Plain CT scan on POD 76 revealed displaced fractures of the right first rib neck (blue arrow) and the left first rib head (red arrow). **C** Plain CT scan before CABG. Compared to this image, the bone fragment of the left first rib head (**B**, red arrow) was shown to be displaced 3 mm anteriorly, which suggested that the fragment affected the stellate ganglion in the sympathetic trunk

A 69-year-old woman with a history of percutaneous coronary intervention and a total abdominal hysterectomy with bilateral salpingo-oophorectomy had chest pain on exertion. Coronary angiography showed severe triple vessel disease. The SYNTAX score was 30, which favored CABG over percutaneous coronary intervention. She underwent off-pump CABG without anti-platelet respite. A pillow was placed under the back adjacent to the shoulder bones. A median sternotomy was performed, and the split sternums were widened using a sternal retractor (IMR15-710-J; Getinge, Gothenburg, Sweden) (Fig. 1A, B). Bilateral internal thoracic arteries (ITAs) were harvested with a Harmonic® scalpel (Johnson and Johnson, NJ, USA). A saphenous vein graft was also harvested. A triple CABG was performed. The respirator was withdrawn on postoperative day (POD) 1. Her recovery was uneventful, but she complained of left shoulder pain after the surgery. She also had anhidrosis involving the left face and hyperhidrosis involving the right face from POD 6. The pain persisted and was treated with acetaminophen. The patient had such a severe allergy to a contrast agent that myocardial scintigraphy was substituted for a postoperative coronary CT. A plain CT and MRI were not performed. The patient was discharged on POD 16. After discharge, in addition to anhidrosis involving the left face, left ptosis and blurry vision appeared, for which she saw a neurologist in our hospital on POD 48 (Fig. 2A). Miosis could not be clearly confirmed. She was diagnosed with Horner’s syndrome. A head MRI revealed no lesions around the medulla oblongata. The symptoms persisted and a plain CT scan on POD 76 revealed displaced fractures of the bilateral first ribs and left second rib (Fig. 2B), which were thought to be caused by rib retraction during the CABG. The bone fragment of the left first rib head was displaced 3 mm anteriorly compared to the left first rib head before surgery (Fig. 2C), which suggested that the fragment affected the stellate ganglion in the sympathetic trunk. The patient had regular follow-up evaluations. At the 6-month follow-up evaluation, the anhidrosis persisted, but the ptosis and blurry vision improved, and the miosis was not confirmed.

To elucidate the relationship between Horner’s syndrome and the first rib fracture due to CABG using ITA in our hospital, we retrospectively researched 139 patients who underwent CABG using an ITA from January 2022 to February 2024 who were followed by coronary or plain CT scans within 2 months after CABG (Table 1). In all cases, the same sternal retractor (IMR15-710-J) was used. The bilateral ITAs, LITA, and RITA were used in 91, 44, and 4 patients, respectively. The types of rib fractures were categorized as displaced or non-displaced (infraction fracture), and the sites of rib fractures were divided into the head, neck, tubercle, and body. Among all patients who sustained rib fractures, no patient sustained more than one fracture per rib. Among 95 patients in whom the RITA was used, 31 (32.6%) patients sustained a right first rib fracture, and among 135 patients in whom the LITA was used, 37 (27.4%) patients sustained a left first rib fracture. Of both ribs, a fracture of rib body was most frequent (right, 54.8%; left, 56.8%). Although 8 (5.8%) of 139 patients sustained the first rib head fracture, only the patient described herein (0.72%) developed Horner’s syndrome.

**Table 1:** The first rib fracture after CABG using the ITA in our hospital (2022.1∼2024.2)

			Right				Left			
	Harvested ITA		95				135			
	Site		Head	Neck	Tubercle	Body	Head	Neck	Tubercle	Body
First rib fracture	Type	Displaced	0	0	1	1	1 (this case)	0	0	8
		Non-displaced (infraction)	3	10	0	16	4	5	6	13
	Total		31				37			

*ITA* Internal thoracic artery

## Discussion

Horner’s syndrome results from damage to the ipsilateral oculosympathetic pathway. Lung, breast, and mediastinum tumors, as well as neck injuries may cause the syndrome due to direct compression of the oculosympathetic pathway [5]. However, cardiovascular surgery can also cause Horner’s syndrome. Only six case reports [1, 6–10] of Horner’s syndrome due to cardiovascular surgery were identified on a search of the literature (Table 2), which may in part be because the symptoms of Horner’s syndrome do not always appear simultaneously and are sometimes too mild to be noticed. Left-sided Horner’s syndrome was attributed to CABG in three cases [1, 6, 10] and the current case, and left lateral thoracotomy operation in two case [7, 8]. In most cases, the firstly recognized Horner’s symptom was ptosis and the onset day was within 2 days after the operation. Generally　speaking, Horner’s syndrome caused by trauma is immediately diagnosed after a traumatic event, but the symptoms can be shown in a delayed manner [11], as in the current case. In some cases, the symptoms resolved spontaneously or with medication.

**Table 2:** Case reports of Horner’s syndrome caused by cardiovascular surgery

Author and year of publication	Age (years)	Sex (M/F)	Affected side	Surgery	Incision	Used ITA	The first symptom and the onset day	Findings on radiology	Treatment and course
Imamaki et al. in 2006 [1]	70	F	Left	Off-pump CABG	Median sternotomy	BITA	Ptosis on POD2	No data	Spontaneous remission 1 month after the surgery
Murakami et al. in 2007 [6]	77	F	Left	MAP + CABG	Median sternotomy	LITA	Ptosis, miosis, and enophthalmos on POD2	Fracture of left 1st rib on chest X-ray	Spontaneous remission 6 months after the surgery
Tsuchiya et al. in 2013 [7]	0 (1 month)	F	Left	PA banding + PDA closure	Left lateral thoracotomy	No use	Ptosis just after the operation	No data	Transconjunctival resection of Muller muscle 1.5 years after the surgery
Nasser et al. in 2015 [8]	0 (9 months)	F	Left	Division of vascular ring	Left lateral thoracotomy	No use	Ptosis, miosis, and enophthalmos on POD2	No data	No remission 7 days after the surgery
Aslankurt et al. in 2021 [9]	9	M	Right	VSD, aortic and mitral valve repair	Median sternotomy	No use	Ptosis on unknown day	No data	No remission 4 months after the surgery
Gopinath et al. in 2021 [10]	56	M	Left	CABG	Median sternotomy	No data	Unknown symptom on POD1	Infraction fracture of left 1st rib head on CT	Remission with Antiimmflammatories and steroids
This case in 2024	69	F	Left	Off-pump CABG	Median sternotomy	BITA	Anhidrosis on POD6	Displaced fracture of 1st rib and left 2nd rib on plain CT	Anhidrosis remained, but ptosis improved 6 months after the surgery

*CABG* Coronary artery bypass grafting, *MAP* Mitral annuloplasty, *PA* Pulmonary artery, *PDA* Patent ductus arteriosus, *ITA* Internal thoracic artery, *BITAs* Bilateral internal thoracic arteries

**Fig. 3:**
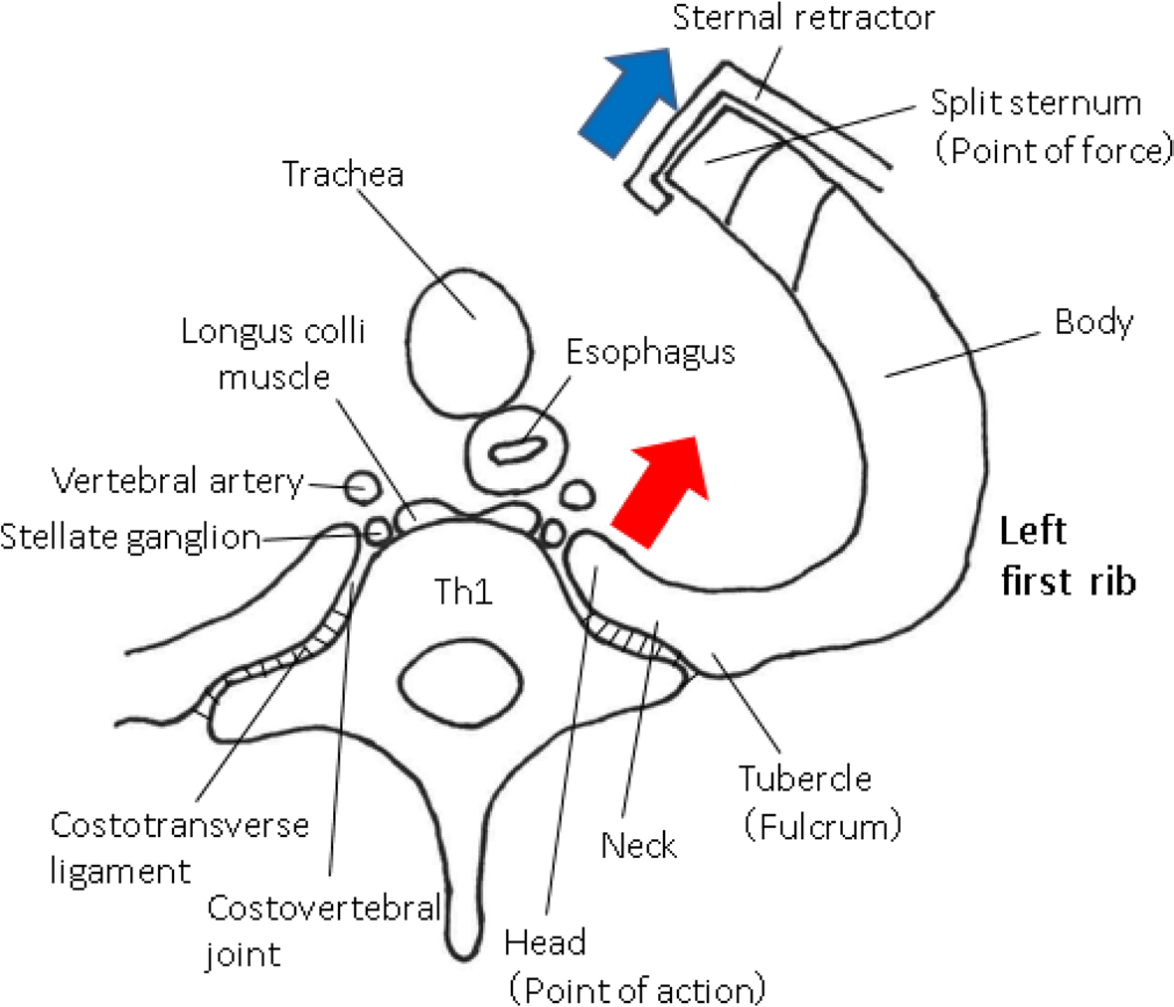
Schema for the mechanism underlying rib fracture. Forceful retraction (blue arrow) can exert excessive work according to the principle of leverage (red arrow), leading to a fracture at the point of action (rib head). If the rib is fragile, the power of excessive retraction may be conducted to the point of action, resulting in disruption of the leverage principle and fracture between the fulcrum (rib tubercle) and point of force (split sternum)

A sternal retractor, especially one for harvesting the ITAs, can cause rib fractures. Kimura [12] reported up to five fractures of the left upper rib after CABG using the left ITA (LITA). Rib retraction for harvesting ITAs after a median sternotomy exerts leverage on ribs like a nail puller. Specifically, the rib tubercle, split sternum, and rib head are the fulcrum, point of force, and point of action, respectively (Fig. 3). The rib head and neck are fixed by the costovertebral joint and costotransverse ligament, respectively. A sternal retractor for ITA harvesting is often placed at an upper position across the sternal angle. Forceful retraction during harvesting an ITA can exert excessive work according to the principle of leverage, leading to fracture of the point of action (head of the first rib). If the rib is fragile, the power of excessive retraction may focus on the point of action, resulting in disruption of the principle of leverage and a fracture between the fulcrum (rib tubercle) and the point of force (split sternum). The rib fracture on CABG will incur more bleeding because perioperative anti-platelet drug therapy is a cornerstone of CABG. A displaced fracture and the ensuing bleeding could lead to injury of the surrounding organs and a hemothorax.

**Fig. 4:**
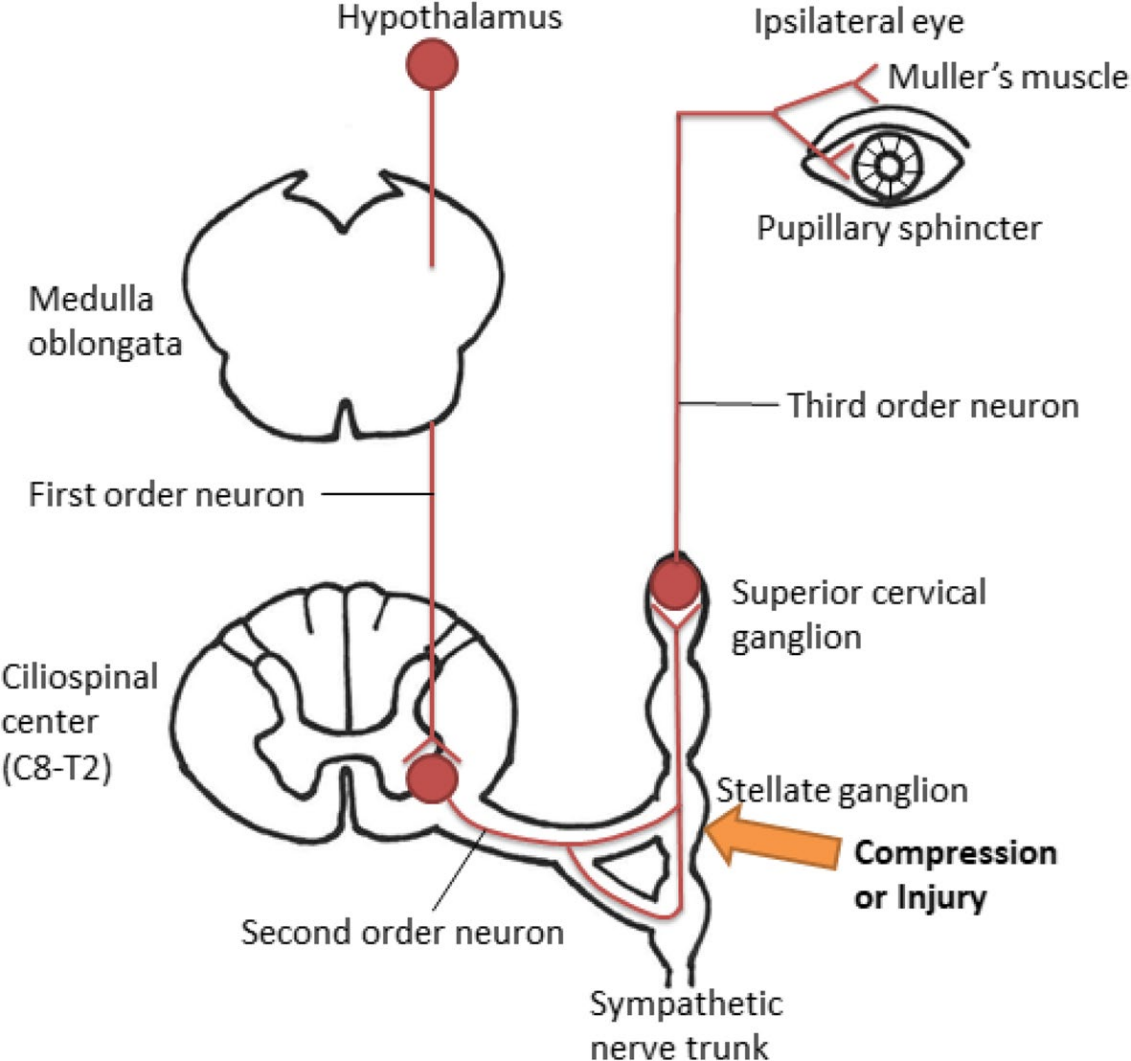
Schema involving the mechanism underlying Horner’s syndrome due to fracture of the first rib head. A bone fragment of the first rib head and subsequent hematoma can compress or injure the stellate ganglion (orange arrow), which is a second-order neuron of the oculosympathetic pathway, leading to Horner’s syndrome. Copyright^©^ 2011, Medic Media. Reproduced with permission from Institute for Health Care Information Sciences ed. *Medical Disease: An Illustrated Reference Guide. vol. 7 Neurology and Neurosurgery*. 1st ed. Tokyo, Japan: Medic Media; 2011 [15]

A dislocation fracture of the costovertebral joint has been reported to cause injury to the intercostal artery and azygos vein, leading to a hematoma [13]. In the same way, a bone fragment of the first rib and subsequent hematoma is considered to directly or indirectly compress brachial nerve plexus and stellate ganglion [3, 4, 12] (Fig. 4). The stellate ganglion, which is a second-order neuron of the oculosympathetic pathway, is located on the ventral surface of the first rib head [14] (Fig. 3). The left stellate ganglion is more densely surrounded than the right ganglion by the vertebral artery, esophagus, longus colli muscle, vertebral body, and left rib head, which might explain why Horner’s syndrome occurs more often on the left side after open heart surgery. In addition, the LITA is used more frequently than the right ITA (RITA) during CABG for its good patency.

Horner’s syndrome complicates all open heart surgery in 0.6–1.7% of cases [3, 16–18] and 0.2–7.7% of CABG cases [19–21]. We retrospectively reviewed 139 CABG cases, among which 8 patients (5.8%) sustained the first rib head fracture (Table 1). Among those 8 patients, only the patient with displaced fracture described herein (0.72%) developed Horner’s syndrome, and the other 7 patients with non-displaced fracture (infraction fracture) did not develop Horner’s syndrome. This can not only graphically but also statistically prove that the displaced bone fracture of the first rib head and the ensuing inflammation and hematoma directly compress or injure the left stellate ganglion and lead to Horner’s syndrome.

Horner syndrome after cardiovascular surgery may be a self-limiting complication based on the outcomes of 7 patients (Table 2), including the patient described herein. This finding can be in part because the inflammation and hematoma surrounding the fracture improved over time and the injured neuron recovered spontaneously. However, we should take Horner syndrome into account as a preventable complication. Minimum sternal widening and careful manipulation of a sternal retractor may prevent iatrogenic Horner’s syndrome [6]. The incidence of first rib fractures due to median sternotomy has been reported to decrease when the sternal retractor is placed at a lower position [3].

**Fig. 5:**
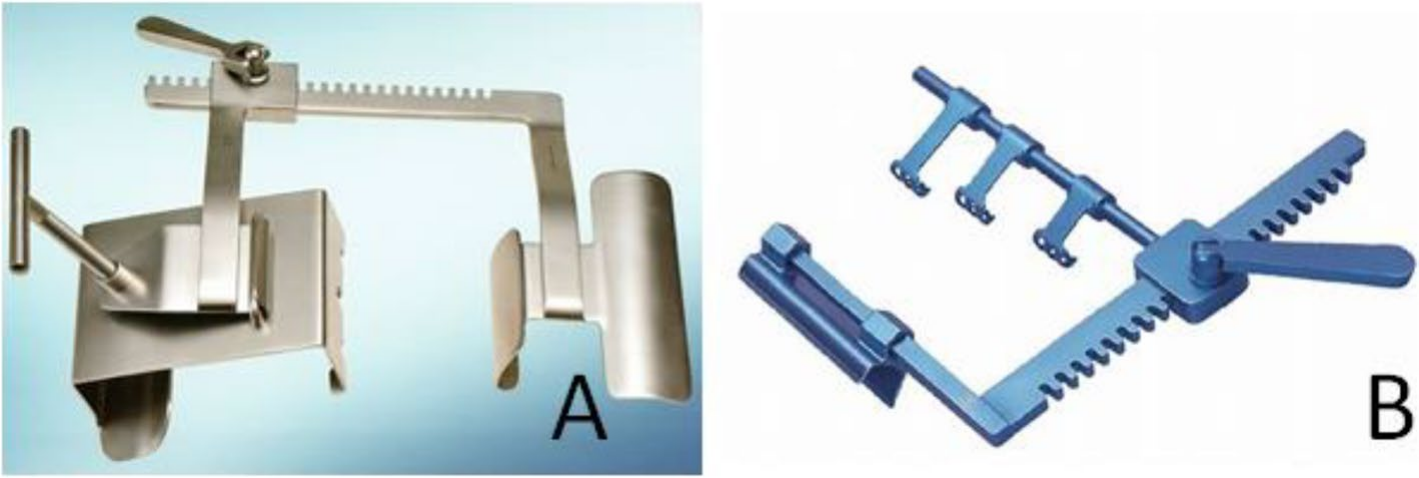
Two types of sternal retractor for harvesting the ITAs. **A** Takedown pattern retractor (IMR15-710-J; Getinge, Gothenburg, Sweden). **B** French pattern retractor (SCT70026 Sternal-IMA Retractor, M A Corporation, Chiba, Japan)

From the viewpoints of the retraction blade, a sternal retractor for harvesting the ITAs is mainly divided into two types: Takedown pattern retractor and French pattern retractor (Fig. 5). There are no reports comparing the two retractors in the literature. A sternal retractor can be equipped with foil load sensors to measure the force distribution over the retractor blades [2]. Therefore, further research for the relation among retraction force, chest opening width, harvesting time, and orthopedic and neurological complications for each retractor is expected, which may lead to a decrease in the complications by retractors.

## Conclusion

We should recognize that Horner’s syndrome is one of the complications of cardiovascular surgery, especially CABG. Fracture of the first rib head with a displaced bone fracture is a contributor to ipsilateral Horner’s syndrome. When symptoms of Horner’s syndrome and other neurologic symptoms are noted after cardiovascular surgery, a plain CT examination should be obtained.

## Data Availability

There are no additional data to disclose.

## Abbreviations

CABG: Coronary artery bypass grafting
PCI: Percutaneous coronary intervention
ITA: Internal thoracic artery
POD: Postoperative day
LITA: Left internal thoracic artery
RITAs: Right internal thoracic arteries

## References

[1] Imamaki M, Ishida A, Shimura H, et al. A case complicated with Horner’s syndrome after off-pump coronary artery bypass. Ann Thorac Cardiovasc Surg. 2006;12(2):113–5. 16702932

[2] Aigner P, Eskandary F, Schlöglhofer T, et al. Sternal force distribution during median sternotomy retraction. J Thorac Cardiovasc Surg. 2013;146(6):1381–6. 24075560 10.1016/j.jtcvs.2013.07.075

[3] Vander Salm TJ, Cereda JM, Cutler BS. Brachial plexus injury following median sternotomy. J Thorac Cardiovasc Surg. 1980;80:447–52. 7412350

[4] Grocott HP, Clark JA, Homi HM, et al. “Other” neurologic complications after cardiac surgery. Semin Cardiothorac Vasc Anesth. 2004;8:213–26. 15375481 10.1177/108925320400800304

[5] Kanagalingam S, Miller NR. Horner syndrome: clinical perspectives. Eye Brain. 2015;10(7):35–46. 10.2147/EB.S63633 PMC5398733 28539793

[6] Murakami T, Kato H, Makino Y. A case of Homer’s syndrome after coronary artery bypass graft surgery. Jpn J Cardiovasc Surg. 2007;36(5):273–6.

[7] Tsuchiya A, Sakurai H, Tsuzuki K. Horner syndrome following cardiac surgery in a child. Jpn J Clin Ophthalmol. 2013;67(5):755–7.

[8] Nasser BA, Mesned A, Moazamy YE, et al. Horner’s syndrome after paediatric cardiac surgery: case report and review of the literature. Cardiol Young. 2015;25(3):569–72. 24717921 10.1017/S1047951114000456

[9] Aslankurt M, Aslan L. Horner’s syndrome secondary to heart surgery in a pediatric patient. Saudi J Ophthalmol. 2020;34(4):303–5. 34527878 10.4103/1319-4534.322597 PMC8409344

[10] Gopinath P, Seetharaman C, Senthilkumar E, et al. Postoperative Horner’s syndrome following CABG - an unusual complication: case report. Eurorad; 2021. Case 17545. https://www.eurorad.org/case/17545. Accessed 4 Sep 2024.

[11] Ryu S, Won S, Lee SY, et al. Delayed Horner’s syndrome after multiple penetrating stab injury of the neck. Ear Nose Throat J. 2022:1455613221125920. https://journals.sagepub.com/doi/epub/10.1177/01455613221125920. Accessed 4 Sep 2024. 10.1177/01455613221125920 36053894

[12] Kimura M, Yoshimura H, Kohara N. Lower trunk brachial plexopathy due to hematoma following median sternotomy: a case report. Rinsho Shinkeigaku. 2020;60(11):758–61. 33115990 10.5692/clinicalneurol.cn-001437

[13] Takizawa K, Suzuki S, Ito S, et al. Vascular injury associated with fracture dislocation of a left costovertebral joint–a case of traumatic subcostohemiazygos fistula. Nippon Igaku Hoshasen Gakkai Zasshi. 1988;25(48):579–83. 3412873

[14] Totoki T, Morimoto M, Taniguchi Y, et al. Anatomy of the stellate ganglion. J Jpn Soc Pain Clin. 1994;1:3–11.

[15] Institute for Health Care Information Sciences, ed. Medical disease: an illustrated reference guide. vol. 7. In: Neurology and neurosurgery. 1st ed. Tokyo: Medic Media; 2011. p. 208.

[16] Benecke R, Klingelhofer J, Rieke H, et al. Manifestations and course of brachial plexus injury following median sternotomy. Nervenarzt. 1988;59:388–92. 3261396

[17] Rieke H, Benecke R, DeVivie ER, et al. Brachial plexus lesions following cardiac surgery with median sternotomy and cannulation of the internal jugular vein. J Cardiothorac Anesth. 1989;3:286–9. 2562481 10.1016/0888-6296(89)90109-9

[18] Vahl CF, Carl I, Muller-Vahl H, et al. Brachial plexus injury after cardiac surgery. The role of internal mammary artery preparation: a prospective study on 1000 consecutive patients. J Thorac Cardiovasc Surg. 1991;102:724–9. 1682532

[19] Lederman RJ, Breuer AC, Hanson MR, et al. Peripheral nervous system complications of coronary artery bypass graft surgery. Ann Neurol. 1982;12:297–301. 6291447 10.1002/ana.410120315

[20] Shaw PJ, Bates D, Cartlidge NE, et al. Neuroophthalmological complications of coronary artery bypass graft surgery. Acta Neurol Scand. 1987;76:1–7. 3498286 10.1111/j.1600-0404.1987.tb03535.x

[21] Barbut D, Gold JP, Heinemann MH, et al. Horner’s syndrome after coronary artery bypass surgery. Neurology. 1996;46:181–4. 8559370 10.1212/wnl.46.1.181

